# Entropy-Regularized Attention for Explainable Histological Classification with Convolutional and Hybrid Models

**DOI:** 10.3390/e27070722

**Published:** 2025-07-03

**Authors:** Pedro L. Miguel, Leandro A. Neves, Alessandra Lumini, Giuliano C. Medalha, Guilherme F. Roberto, Guilherme B. Rozendo, Adriano M. Cansian, Thaína A. A. Tosta, Marcelo Z. do Nascimento

**Affiliations:** 1 Department of Computer Science and Statistics (DCCE), São Paulo State University (UNESP), Rua Cristóvão Colombo, 2265, São José do Rio Preto 15054-000, São Paulo, Brazil; guilherme.botazzo@unesp.br (G.B.R.); adriano.cansian@unesp.br (A.M.C.); 2 Department of Computer Science and Engineering, University of Bologna, Via dell’Università 50, 47522 Cesena, Italy; alessandra.lumini@unibo.it; 3 WZTECH NETWORKS, Avenida Romeu Strazzi (Room 503-B), 325, São José do Rio Preto 15084-010, São Paulo, Brazil; giuliano@wztech.com.br; 4 Department of Informatics Engineering, Faculty of Engineering, University of Porto, Dr. Roberto Frias, sn, 4200-465 Porto, Portugal; guilhermefroberto@gmail.com; 5 Science and Technology Institute, Federal University of São Paulo (UNIFESP), Avenida Cesare Mansueto Giulio Lattes, 1201, São José dos Campos 12247-014, São Paulo, Brazil; tosta.thaina@unifesp.br; 6 Faculty of Computer Science (FACOM), Federal University of Uberlândia (UFU), Avenida João Naves de Ávila 2121, Bl.B, Uberlândia 38400-902, Minas Gerais, Brazil; marcelo.nascimento@ufu.br

**Keywords:** attention branches, CAM Fostering, convolutional neural networks, vision transformers, Grad-CAM, histological images

## Abstract

Deep learning models such as convolutional neural networks (CNNs) and vision transformers (ViTs) perform well in histological image classification, but often lack interpretability. We introduce a unified framework that adds an attention branch and CAM Fostering, an entropy-based regularizer, to improve Grad-CAM visualizations. Six backbone architectures (ResNet-50, DenseNet-201, EfficientNet-b0, ResNeXt-50, ConvNeXt, CoatNet-small) were trained, with and without our modifications, on five H&E-stained datasets. We measured explanation quality using coherence, complexity, confidence drop, and their harmonic mean (ADCC). Our method increased the ADCC in five of the six backbones; ResNet-50 saw the largest gain (+15.65%), and CoatNet-small achieved the highest overall score (+2.69%), peaking at 77.90% on the non-Hodgkin lymphoma set. The classification accuracy remained stable or improved in four models. These results show that combining attention and entropy produces clearer, more informative heatmaps without degrading performance. Our contributions include a modular architecture for both convolutional and hybrid models and a comprehensive, quantitative explainability evaluation suite.

## 1. Introduction

Deep learning models, particularly convolutional neural networks (CNNs) [1,2] and vision transformers (ViTs) [3], have achieved state-of-the-art performance in a variety of visual recognition tasks [4,5]. These advances have enabled the development of computational systems with substantial impact in sensitive and complex domains such as healthcare, where the automated analysis of histological images has emerged as a promising diagnostic aid [6,7,8,9,10,11].

Histopathological analysis plays a central role in the diagnosis of diseases affecting biological tissues. This process involves collecting tissue fragments, staining them using protocols such as hematoxylin and eosin (H&E), and interpreting the resulting slides under a microscope to identify morphological anomalies [12]. Although essential, this task is time-consuming and subject to inter- and intra-observer variability, relying heavily on the experience and judgment of specialists [13]. In this context, the integration of machine learning models, especially CNNs and ViTs, into the histological workflow can help improve diagnostic efficiency, consistency, and scalability [14,15,16,17,18,19].

However, despite their high predictive performance, these models often suffer from limited interpretability, resulting in reduced transparency of their internal decision-making processes and hindering their adoption in clinical practice [20,21]. This lack of interpretability raises concerns about trust, accountability, and clinical validation, which are especially critical in the medical domain [22]. In response to this challenge, the field of explainable artificial intelligence (XAI) has grown rapidly, focusing on techniques that make model predictions more understandable and trustworthy to human experts [23,24,25]. Among the most widely adopted post hoc XAI techniques is gradient-weighted class activation mapping (Grad-CAM) [26], which generates heatmaps indicating the regions of the input image that most influenced the model’s output. In the case of ViTs, attention rollout [27] is frequently used to combine attention scores from multiple layers and heads into a unified visualization. These strategies allow for visual verification of model focus and relevance, serving as a bridge between model outputs and human reasoning.

In parallel to post hoc explanations, several neural network architectures have been designed to improve explainability intrinsically [28,29,30,31]. The attention branch network (ABN) [32] augments convolutional backbones with a dedicated attention branch that guides the network toward relevant features during training, thereby enhancing the informativeness of generated heatmaps. Likewise, the explainable convolutional neural network (XCNN) [33] leverages an encoder–decoder structure to generate and refine attention maps, supported by a discriminator that encourages fidelity and relevance in the learned explanations. More recently, strategies such as CAM Fostering have introduced the use of entropy to regulate the quality of class activation maps [34]. By penalizing low-entropy maps, which are often associated with overly concentrated or overly diffuse attention, this technique encourages the model to generate activation maps that are both spatially diverse and semantically informative. Such regularization has shown promise in improving not only the interpretability but also the generalization of deep learning models.

Despite these advances, there remain important gaps in the literature. First, relatively few explainability strategies have been developed and validated specifically for histological images, which pose unique challenges due to their heterogeneous textures, multi-scale structures, and subtle morphological variations [13,35]. Second, the evaluation of most XAI methods still relies primarily on qualitative metrics, particularly subjective visual inspection of explanation maps [36]. This limits reproducibility and comparability between studies. The development of quantitative metrics capable of objectively assessing the quality of explanations is thus essential for establishing more rigorous evaluation protocols. In addition, although transformer-based models are increasingly used in medical imaging tasks such as segmentation and classification [37,38,39], their potential to produce rich and interpretable explanations has not yet been fully explored. Given their ability to model global contextual relationships via self-attention [3], ViTs may offer significant advantages over CNNs in tasks involving spatially dispersed or subtle diagnostic patterns, as commonly found in histological samples.

To address these limitations, this study proposes a novel explainable model architecture that integrates the attention supervision of the ABN with entropy-based regularization via the CAM Fostering technique. The resulting model is designed to be modular and adaptable, allowing the integration of various neural backbones, including both convolutional and hybrid architectures. In this work, we evaluate our approach using six prominent models, ResNet-50, DenseNet-201, EfficientNet-b0, ResNeXt-50, ConvNeXt, and CoatNet-small, trained on five H&E-stained histological datasets. Thus, for each configuration, we generate explanations using Grad-CAM and assess them using a robust set of quantitative metrics: coherence, complexity, confidence drop, and ADCC (Average DCC), which is the harmonic mean of the three. This evaluation framework enables a comprehensive and objective assessment of how attention and entropy mechanisms contribute to explanation quality across architectures and datasets.

The main contributions of this work are as follows:

- A modular explainable architecture combining attention mechanisms and entropy-based regularization, compatible with convolutional and hybrid models and capable of enhancing the quality and relevance of visual explanations in histological image classification;
- A systematic evaluation of attention and entropy mechanisms across six neural network backbones and five histological datasets;
- A quantitative evaluation framework based on well-defined metrics to objectively assess the quality of visual explanations generated by deep learning models.

## 2. Materials and Methods

This section describes the main steps of the proposed methodology, which combines a modified ABN architecture with the CAM Fostering strategy to improve the interpretability of Grad-CAM explanations across different models. The first step consisted of dividing five histological image datasets using the hold-out strategy [40]. In this case, each dataset was divided into a 70/15/15 ratio, in which 70% of the dataset was dedicated to training, 15% to validation, and 15% to testing.

In the next step, six widely adopted architectures, ResNet-50 [2], DenseNet-201 [41], EfficientNet-B0 [42], ResNeXt-50 [43], ConvNeXt [44], and CoatNet-small [45], were selected based on their frequent use in histological image analysis tasks [46,47,48,49,50,51]. Each model was trained with and without the proposed modification, using the training sets. The selection of the best training across epochs was guided by the highest F1-score in the validation set.

**Figure 1:**
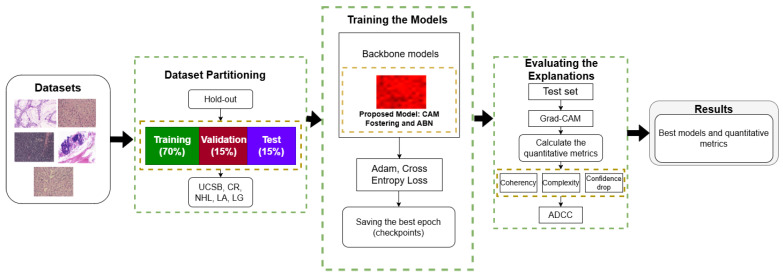
Proposed methodology integrating ABN and CAM Fostering techniques.

For the final step, after training, Grad-CAM was used to generate visual explanations for the test set. These explanation maps were quantitatively evaluated using a set of metrics designed to assess different aspects of explanation quality: coherence, complexity, confidence drop, and average DCC (ADCC), which aggregates the others into a single score [52]. It should be noted that although CoatNet-small is a hybrid architecture that incorporates transformer layers, Grad-CAM remains applicable due to its internal convolutional structure [53]. An overview of the complete methodology is illustrated in Figure 1.

### 2.1. Datasets

This study employed five histological image datasets, composed of static images, covering four tissue types, all stained with H&E. The first dataset (UCSB) contains 58 breast cancer samples provided by the University of California at Santa Barbara [54], categorized into benign (38) and malignant (20) classes.

The second dataset (CR) comprises 165 colorectal tissue images [55], split into benign (74) and malignant (91) cases. Images were acquired using a Zeiss MIRAX MIDI Slide Scanner at a resolution of 0.620 μm, corresponding to 20× magnification. It is important to note that, despite the use of a slide scanner to obtain the images, all the samples in this dataset are static, so it was not necessary to carry out any pre-processing on them.

The third dataset (NHL) was released by the National Cancer Institute in collaboration with the National Institute on Aging [56]. It contains 173 samples of non-Hodgkin’s lymphomas across three classes: mantle cell lymphoma (MCL, 99), follicular lymphoma (FL, 62), and chronic lymphocytic leukemia (CLL, 12). These images were captured using a Zeiss Axioscope microscope at 20× magnification and an AxioCam MR5 camera, producing uncompressed RGB images with a resolution of 1388 × 1040 pixels and 24-bit color depth.

The fourth and fifth datasets were obtained from the Atlas of Gene Expression in Mouse Ageing Project (AGEMAP) [57], using a Carl Zeiss Axiovert 200 microscope at 40× magnification. The fourth dataset (LG) consists of 265 liver tissue images from calorie-restricted rats (150 male, 115 female). The fifth dataset (LA) includes 529 liver images obtained from rats under an ad libitum diet, grouped by age: one month (100), six months (115), 16 months (162), and 24 months (152).

**Figure 2:**
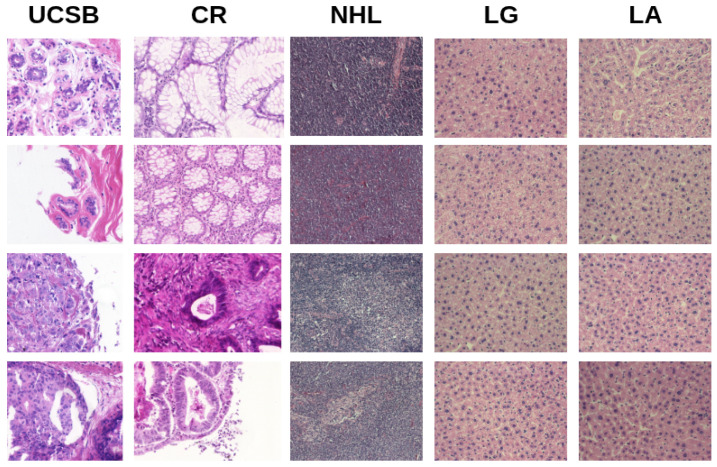
Representative histological samples from each dataset.

Figure 2 shows sample images from each dataset, and Table 1 summarizes their main characteristics.

In this investigation, due to the substantial staining variability among the histological datasets used, no explicit stain normalization techniques were applied [58,59]. Instead, the methodology deliberately preserved the original color distribution of each dataset (UCSB, NHL, CR, LG, and LA) to evaluate the robustness and adaptability of the proposed architecture in real-world scenarios. This decision aimed to ensure that the interpretability results would reflect performance under naturally heterogeneous staining conditions, as often encountered in clinical settings.

### 2.2. Proposed Models

A modified architecture based on the ABN [32] was developed to enhance model explainability through the integration of the CAM Fostering mechanism [34]. This combination allows for improved attention supervision by generating activation maps that are semantically meaningful and spatially informative. The architecture was structured into three main components: a feature extractor, an attention branch, and a perception branch. The attention branch was responsible for producing intermediate attention maps that guided the learning process, while the CAM Fostering mechanism was incorporated during training as a regularization term. By computing the entropy of the attention maps, this mechanism penalized distributions that were either overly concentrated or excessively diffuse, encouraging a balanced and information-rich representation.

**Figure 3:**
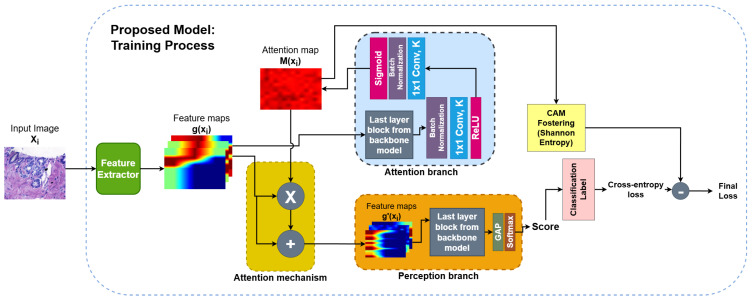
Training process schematic of proposed method: feature extractor, attention branch, and perception branch with CAM Fostering.

In addition, the proposed model was instantiated using six backbone architectures: ResNet-50, DenseNet-201, EfficientNet-b0, ResNeXt-50, ConvNeXt, and CoatNet-small. It is important to note that the proposed model can use other networks as backbones, but these models were chosen due to their architectural diversity and relevance in the context of histological image classification [6,7,14,60,61,62,63,64]. Each modified backbone was trained and evaluated independently to assess the general applicability of the proposed explainability-enhancing strategy. A schematic overview of the proposed architecture is shown in Figure 3.

#### 2.2.1. Feature Extractor

The feature extractor is the first component of the proposed model. This module is composed of all the intermediate and convolutional layers of each backbone architecture (ResNet-50, DenseNet-201, EfficientNet-b0, ResNeXt-50, ConvNeXt, and CoatNet-small), excluding the final classification-specific blocks. Its function is to transform the input image $X_{i}$ into a set of feature maps $g(X_{i})$ that capture the hierarchical spatial patterns inherent in histological images, such as texture granularity, cellular morphology, and tissue architecture [33]. These feature maps represent a rich and semantically dense encoding, which supports both the interpretability and classification tasks of the model.

The extracted feature maps are then simultaneously forwarded to the two main modules of the architecture: the attention branch and the perception branch. The attention branch is designed to generate spatial attention maps $M(X_{i})$ that highlight class-relevant regions. These maps are subsequently used by the attention mechanism to guide feature refinement, and by the CAM Fostering strategy to regularize the distribution of attention through entropy-based constraints. This dual usage promotes consistency between the areas of the image that drive the model’s predictions and those presented as explanations. A detailed description of the attention branch is provided in the next subsection.

In parallel, the perception branch applies an attention mechanism that modulates the original feature maps using the attention maps, yielding a refined representation $g^{\prime }\left(X_{i}\right)$ that focuses on diagnostically relevant areas. This design encourages a functional alignment between explanation and decision-making, addressing known limitations in previous XAI approaches in medical imaging, which often treat interpretability as a post hoc or external process [22,36].

#### 2.2.2. Attention Branch

The attention branch received as input the feature maps $g(X_{i})$ generated by the feature extractor and produced a spatial attention map $M(X_{i})$. Subsequently, this map was used to modulate the classification characteristics and calculate the entropy term in the CAM Fostering strategy, enhancing the interpretability of the model during training [65].

Structurally, the attention branch consisted of the final convolutional block of each backbone, which produced a tensor of dimensions K×w×h, where *K* is the number of channels and w×h the spatial resolution. This tensor was passed through a sequence of batch normalization, a 1×1 convolutional layer, and a ReLU activation, reducing the dimensionality to a single-channel intermediate map. A second normalization and activation sequence, comprising another batch normalization layer, a 1×1 convolution, and a sigmoid function, was applied to generate the final attention map $M(X_{i})$, constrained to the interval [0,1].

Importantly, the proposed attention branch differs from the original ABN formulation by excluding the auxiliary classification layer traditionally attached to the attention map. This modification was essential to support the CAM Fostering mechanism, which leverages the attention map solely as a spatial prior, without requiring parallel classification outputs.

#### 2.2.3. Attention Mechanism

The attention mechanism implemented in the perception branch followed the formulation established in the original ABN model [32], where the attention map $M(X_{i})$ was used to modulate the original feature maps $g(X_{i})$, producing a refined set of features $g^{\prime }\left(X_{i}\right)$. This process emphasizes regions deemed relevant for the prediction, enhancing the interpretability and effectiveness of the classification output. The mechanism is defined in Equation (1):

(1) $$g^{\prime }\left(X_{i}\right)=\left(g\left(X_{i}\right)\times M\left(X_{i}\right)\right)+g\left(X_{i}\right)$$

This formulation combines element-wise attention with residual learning. The attention term selectively enhances salient regions, while the residual connection preserves the full original feature context, promoting representational stability and improving gradient flow during training.

#### 2.2.4. Perception Branch

The perception branch was responsible for generating the model’s final classification output. It received as input the enhanced feature maps $g^{\prime }\left(X_{i}\right)$ produced by the attention mechanism, which integrated both the original feature representation and the spatial guidance from the attention maps. This branch reused the final convolutional block of the original backbone architecture, preserving the semantic abstraction inherent in its design.

Following this block, a global average pooling (GAP) layer was applied to compress the spatial dimensions of each feature map into a single scalar value. This operation transformed the K×w×h tensor into a *K*-dimensional vector, where each value represented the global activation of a corresponding channel. This representation was subsequently passed through a softmax activation function to yield normalized class probabilities.

The adoption of GAP, in the place of the fully connected layer used in the original ABN model, served a dual purpose. First, it preserved the spatial correspondence of the convolutional features, which is crucial for maintaining interpretability, by avoiding the loss of spatial localization cues [66]. Second, it reduced the number of trainable parameters, thereby minimizing the risk of overfitting. This architectural choice ensured that the discriminative regions identified by the attention mechanism remained directly linked to the final classification outcome, reinforcing the model’s capacity to produce spatially coherent and clinically relevant explanations [67].

#### 2.2.5. CAM Fostering

To further enhance the interpretability of the model, the CAM Fostering strategy [34] was integrated as an auxiliary mechanism during training. This approach introduces an information-theoretic constraint on the attention maps, encouraging the generation of activation patterns that are neither overly sparse nor excessively diffuse.

The mechanism operates by computing the Shannon entropy ce of the attention map $M(X_{i})$, which quantifies the diversity of activations across the spatial domain. Attention maps with highly uniform activations exhibit low entropy, indicating poor localization capacity, while maps with diverse spatial responses exhibit higher entropy, suggesting richer explanatory content. The entropy ce is formally defined in Equation (2):

(2) $$ce\left(M\left(X_{i}\right)\right)=-\sum_{ij}M{\left(X_{i}\right)}_{ij}\ln M{\left(X_{i}\right)}_{ij}$$

The indices ij span the two-dimensional spatial domain of the attention map. During training, this entropy value was incorporated into the loss function as a regularization term, weighted by the factor $\gamma_{e}\in \left[0,10\right]$. As suggested in the original formulation [34], higher values of $\gamma_{e}$ amplify the influence of the entropy regularization, improving explanation quality at the potential cost of classification accuracy.

The final training objective $l_{n}^{\prime }$ was defined as the original cross-entropy loss $l_{n}$ subtracted by the entropy-weighted regularization term, as shown in Equation (3):

(3) $$l_{n}^{\prime }=l_{n}-\gamma_{e}\cdot ce\left(M\left(X_{i}\right)\right)$$

In this study, CAM Fostering was applied to the attention maps $M(X_{i})$ generated by the attention branch of each model. A regularization factor of $\gamma_{e}=10$ was used to maximize the regularization effect, ensuring the generation of more spatially diverse and informative attention maps. The cross-entropy loss function [68] remained the primary optimization criterion, while the CAM Fostering term was used as a complementary constraint to balance classification performance with explanation quality.

### 2.3. Dataset Partitioning and Experimental Setup

To ensure consistent and unbiased evaluation of each model’s classification and explanatory capacity, a standardized dataset partitioning strategy was adopted. Each of the five histological datasets was independently divided into training (70%), validation (15%), and test (15%) subsets using a hold-out protocol [40]. Images were randomly assigned to each subset to avoid selection bias and to preserve the original class distributions. This experimental design enabled robust model comparison across architectures and configurations, including the evaluation of explanation quality.

### 2.4. Training Protocol and Optimization Strategy

Each model—ResNet-50, DenseNet-201, EfficientNet-b0, ResNeXt-50, ConvNeXt, and CoatNet-small—was trained in two configurations: (i) as a standard baseline model, and (ii) as a backbone integrated with the proposed attention-based architecture and CAM Fostering regularization.

To accelerate convergence and reduce overfitting, transfer learning was employed [69]. All models were initialized with weights pre-trained on ImageNet [70] and fine-tuned on the histological datasets. Training was performed over 20 epochs, using a batch size of 16 and a learning rate of 0.0001. It is worth noting that training was carried out for each dataset. The Adam optimizer [71] was selected for its adaptive learning dynamics and efficiency in training deep models with limited epochs.

The cross-entropy loss function [68] was used consistently for both the baseline and modified models. For the models incorporating CAM Fostering, the entropy-based regularization term was subtracted from the primary loss during optimization (see Section 2.2.5).

To ensure optimal generalization, a model checkpointing strategy was adopted, whereby the F1-score was calculated on the validation set after each epoch, and the model weights from the epoch with the highest F1-score were retained. This approach prioritized balanced performance, particularly in the presence of class imbalance, and reduced the risk of overfitting and underperformance on minority classes. Moreover, it promoted the learning of features that generalize beyond superficial visual cues such as color intensity or contrast. As a result, the explainability evaluation, based on Grad-CAM and complementary metrics, focused on spatial coherence and semantic alignment, which are inherently more resilient to staining variability and less dependent on color distribution.

### 2.5. Evaluation of Explanations

To quantify the quality of the visual explanations generated by each trained model, a set of complementary metrics was computed using the Grad-CAM outputs on the test datasets. These metrics—coherence, complexity, confidence drop, and average DCC (ADCC)—assess different dimensions of explanation reliability, consistency, and informativeness [52]. Together, they offer a comprehensive evaluation framework for interpretability in the context of medical imaging.

#### 2.5.1. Coherence (CO)

The coherence metric evaluates the stability and internal consistency of an activation map. Given an image *x* classified as class *c*, the activation map $CAM_{c}\left(x\right)$ is considered coherent if it remains unchanged when applied back to the image through element-wise masking, i.e., $CAM_{c}\left(x⊙CAM_{c}\left(x\right)\right)\approx CAM_{c}\left(x\right)$. This property is formally expressed in Equation (4).

(4) $$CAM_{c}\left(x⊙CAM_{c}\left(x\right)\right)=CAM_{c}\left(x\right)$$

To measure this property, the Pearson correlation coefficient is computed between the original and transformed activation maps, as shown in Equation (5). The result is normalized to the interval [0,1], where values closer to 1 indicate higher coherence and robustness of the explanation [72,73,74].

(5) $$Coherence\left(x\right)=\frac{\mathrm{Cov}\left(CAM_{c}\left(x⊙CAM_{c}\left(x\right)\right),\;CAM_{c}\left(x\right)\right)}{\sigma \left(CAM_{c}\left(x⊙CAM_{c}\left(x\right)\right)\right)\;\sigma \left(CAM_{c}\left(x\right)\right)}$$

#### 2.5.2. Complexity (COM)

The complexity metric quantifies the spatial dispersion of the activation map. High-complexity maps tend to activate over broad, diffuse regions, which may hinder clinical interpretability by introducing ambiguity. In contrast, low-complexity explanations that concentrate on compact, diagnostically relevant areas are generally more desirable. To estimate this behavior, the $L_{1}$ norm of the activation map is employed, as formalized in Equation (6).

(6) $$Complexity\left(x\right)={∥CAM_{c}\left(x\right)∥}_{1}$$

Values are bounded in the range [0,1], where lower scores indicate more concise and focused explanations.

#### 2.5.3. Confidence Drop (CD)

The confidence drop measures how much the model’s prediction confidence decreases when restricted to only the regions highlighted by the explanation. Let $y_{c}$ be the prediction score on the full image and $o_{c}$ be the score on the masked input. The metric is defined as follows:

(7) $$ConfidenceDrop\left(x\right)=\frac{\max(0,y_{c}-o_{c})}{y_{c}}$$

Considering that the values are represented in the range [0,1], smaller values indicate that the explanation captures the regions truly responsible for the prediction, preserving confidence under restricted input. Thus, a lower CD implies better faithfulness of the explanation to the model’s internal decision process [75].

#### 2.5.4. Average DCC (ADCC)

To consolidate the performance across the three dimensions above, the ADCC metric computes the harmonic mean of Coherence, 1−Complexity, and 1−ConfidenceDrop:

(8) $$ADCC\left(x\right)=3{\left(\frac{1}{Coherence(x)}+\frac{1}{1-Complexity(x)}+\frac{1}{1-ConfidenceDrop(x)}\right)}^{-1}$$

This metric penalizes any weakness in a single aspect, ensuring that only balanced and informative explanations receive high scores. This metric is represented by values in the interval [0,1], where higher ADCC values indicate that the explanations are consistent, concise, and faithful, which are critical characteristics for trustworthy use in clinical and scientific settings.

### 2.6. Software Packages and Execution Environment

The implementation of the proposed methodology was carried out using the Python programming language, version 3.12.3. Model development and training were conducted using the PyTorch 2.7.0 deep learning framework [76], in combination with the PyTorch-Ignite library [77], which was used to streamline training and evaluation routines. Classification performance metrics were computed using the Scikit-learn library [78], while all calculations related to explanation metrics, such as coherence, complexity, confidence drop, and entropy, were performed using NumPy [79]. In addition, all experiments were executed in a Linux-based environment (kernel version 6.8.0), on a machine equipped with an Intel Core i7-1360H processor, 32 GB of RAM, and an NVIDIA RTX 4050 GPU with 6 GB of dedicated memory.

## 3. Results and Discussion

This section presents a systematic evaluation of the proposed methodology through a three-stage experimental protocol. Each stage was designed to assess distinct aspects of the model’s performance and interpretability, delivering quantitative and qualitative insights into the effectiveness of the introduced architectural modifications.

In the first stage (Section 3.1), the original backbone models, ResNet-50, DenseNet-201, EfficientNet-b0, ResNeXt-50, ConvNeXt, and CoatNet-small, were evaluated using the explainability metrics described in Section 2.5: coherence (CO), complexity (COM), confidence drop (CD), and average DCC (ADCC). This established a baseline for interpretability against which subsequent improvements could be compared. In the second stage (Section 3.2), the proposed architecture, integrating attention supervision and CAM Fostering, was applied to each backbone. The modified models were re-evaluated using the same metrics, allowing us to assess the quantitative impact of the proposed modifications on explanation quality. In the final stage (Section 3.3), a comparative visual analysis was conducted. Grad-CAM heatmaps from the original and modified models were juxtaposed to qualitatively illustrate the interpretability improvements in representative samples from the histological datasets.

### 3.1. Baseline Explainability Assessment

Table 2, Table 3, Table 4, Table 5 and Table 6 report the explainability metrics—coherence (CO), complexity (COM), confidence drop (CD), and average DCC (ADCC)—for the original backbone models evaluated on the five histological datasets. In these results, higher values of CO and ADCC are preferable (↑), while lower values of COM and CD are desirable (↓). It is important to note that all the metrics are represented in percentage format for a better interpretation of the results. These per-dataset evaluations enable a detailed analysis of model interpretability across distinct histological domains, highlighting the influence of architectural characteristics on the quality of saliency-based explanations. These results also serve as a baseline for assessing the interpretability gains achieved by the proposed architecture in subsequent analyses.

Among the evaluated models, CoatNet-small consistently demonstrated high ADCC scores across all datasets, notably achieving top performance on NHL (70.74%), LG (65.44%), and LA (71.60%). This trend indicates a strong alignment between the predicted classes and the spatial regions highlighted by Grad-CAM, suggesting that the model’s hybrid architecture, combining convolutional layers with vision transformer (ViT) blocks, enables more semantically coherent and spatially meaningful explanations. The global receptive fields of ViT layers are particularly beneficial in these histopathological contexts, where relevant structures may be non-contiguous and dispersed across the image [3].

EfficientNet-b0 also performed competitively, especially in datasets with lower variability, such as UCSB (ADCC = 54.33%) and LA (66.84%). Despite being the most compact model in terms of parameters, its compound scaling and architectural efficiency appear to support the generation of stable and interpretable feature hierarchies. This challenges the assumption that model depth alone guarantees better interpretability, highlighting the relevance of multi-scale normalization and efficient design. ResNeXt-50 and ConvNeXt displayed moderate but consistent ADCC values across datasets, particularly excelling on the CR dataset (65.39% and 53.68%, respectively), which may be attributed to their modular architectures and enhanced feature aggregation mechanisms. This behavior suggests a tendency to produce more structured attention over diagnostically relevant regions, although with less spatial precision compared to hybrid models.

In contrast, DenseNet-201, while achieving high coherence in some datasets (for instance, UCSB: CO = 35.08%), generally exhibited lower ADCC values, such as on CR (50.09%) and NHL (42.86%). This suggests that although dense connectivity promotes feature reuse, the lack of explicit attention mechanisms may hinder the model’s ability to generate spatially focused and semantically aligned explanations.

Overall, the results from Table 2, Table 3, Table 4, Table 5 and Table 6 highlight significant variability in the natural explainability of convolutional and hybrid architectures. These differences reinforce the necessity of incorporating mechanisms like attention guidance and entropy-aware regularization to ensure that deep models not only perform well in classification, but also offer transparent and clinically reliable explanations, a crucial aspect in sensitive domains such as medical imaging.

### 3.2. Evaluating Proposed Models

Table 7, Table 8, Table 9, Table 10 and Table 11 present the percentage values of the explainability metrics obtained after applying the proposed model-combining attention supervision with entropy-based regularization (CAM Fostering) to each backbone across all datasets. For each configuration, the tables report the values for coherence (CO), complexity (COM), confidence drop (CD), and the aggregate metric ADCC. In this context, higher values of CO and ADCC (↑) indicate better interpretability, whereas lower values of COM and CD (↓) suggest more concise and confident explanations. Similarly to the results obtained by the backbone models, all the metrics are represented in percentages.

On the UCSB dataset (Table 7), ConvNeXt achieved the highest CO (35.42%) and ADCC (62.69%), along with the lowest CD (7.00), indicating that the proposed strategy enhances interpretability even in small-scale scenarios. EfficientNet-b0 also performed well (ADCC: 61.86%) due to a low CD, despite a higher COM. For the NHL dataset (Table 8), CoatNet-small obtained the highest ADCC (77.90%), supported by a strong CO (40.05%) and minimal COM (0.07%). Moreover, ConvNeXt registered the lowest CD (0.52%), reinforcing its ability to generate confident and stable explanations in visually complex samples. On the CR dataset (Table 9), ResNeXt-50 achieved the highest CO (34.12%) and ADCC (62.77%), with the lowest COM (0.13%), while ResNet-50 achieved the lowest CD (5.74%), suggesting that convolutional architectures benefit notably from entropy-based regularization in simpler visual contexts. In the LG dataset (Table 10), CoatNet-small again led in ADCC (69.14%) and CO (37.71%), while ConvNeXt showed the lowest CD (8.53%), confirming its robustness across heterogeneous tissue morphologies. For the LA dataset (Table 11), CoatNet-small achieved the highest ADCC (75.11%) and CO (36.57%), with ConvNeXt maintaining the lowest CD (15.79%), highlighting its consistency in producing stable attention maps in large-scale, pattern-rich datasets.

Regarding generalization and deployment, the architecture demonstrated robustness across datasets with varying complexity and scale. In low-variability or small-sample scenarios (for instance, UCSB), the interpretability metrics remained stable, and EfficientNet-b0 maintained its classification performance, supporting its suitability in resource-constrained environments. Conversely, on morphologically complex datasets such as NHL and LA, entropy-regularized attention yielded the most substantial interpretability gains, confirming its capacity to generalize under high variability. These findings underscore the practical viability of the proposed solution across diagnostic settings with diverse computational and clinical demands.

In this context, the consistent gains in interpretability across datasets validate the effectiveness of combining attention alignment with entropy regularization, regardless of model architecture or dataset complexity.

#### Summary of Explainability Results: Baseline Versus Proposed Models

Table 12 summarizes the average explainability metrics across all datasets, comparing each backbone in its baseline form and after applying the proposed strategy. The best results for each metric are highlighted in bold.

Considering this comparative overview (Table 12), ResNet-50 showed the most substantial improvement, with the ADCC increasing from 47.72% to 63.37%, representing a gain of 15.65%. This highlights the advantage of incorporating attention alignment and entropy regularization in architectures that lack built-in global context modeling. DenseNet-201 experienced a slight decrease in overall ADCC (from 52.03% to 51.55%) when averaged across all datasets. However, per-dataset analysis reveals improvements on four datasets, particularly on NHL, where the ADCC increased by 14.11%. In addition, the decline on UCSB may be attributed to the dataset’s limited size and variability, which can affect the impact of regularization. Also, EfficientNet-b0’s ADCC dropped marginally by 1.59%, despite gains on UCSB and LG, likely due to its highly optimized design constraining the influence of additional regularization. On the other hand, ResNeXt-50 benefited from the strategy with a 7.05% increase in ADCC, particularly on NHL (+18.01%), suggesting that its modular topology integrates well with the proposed refinements. ConvNeXt’s ADCC improved by 8.74%, with the most notable gain on UCSB (+11.7%). Despite already capturing long-range dependencies, the method further enhanced the model’s interpretability. Finally, CoatNet-small, the strongest baseline, achieved a 7.16% increase in ADCC, reaching 77.90% on NHL, indicating that its hybrid architecture effectively benefited from the attention–entropy regularization. Overall, the proposed strategy consistently enhances interpretability by improving coherence and reducing uncertainty, regardless of architectural design or dataset scale. Moreover, these results also enable the possibility of evaluating the proposed approach with additional backbone models. The observed gains further reinforce its applicability across diverse histological domains and architecture types.

### 3.3. Visual Explainability Analysis

**Figure 4:**
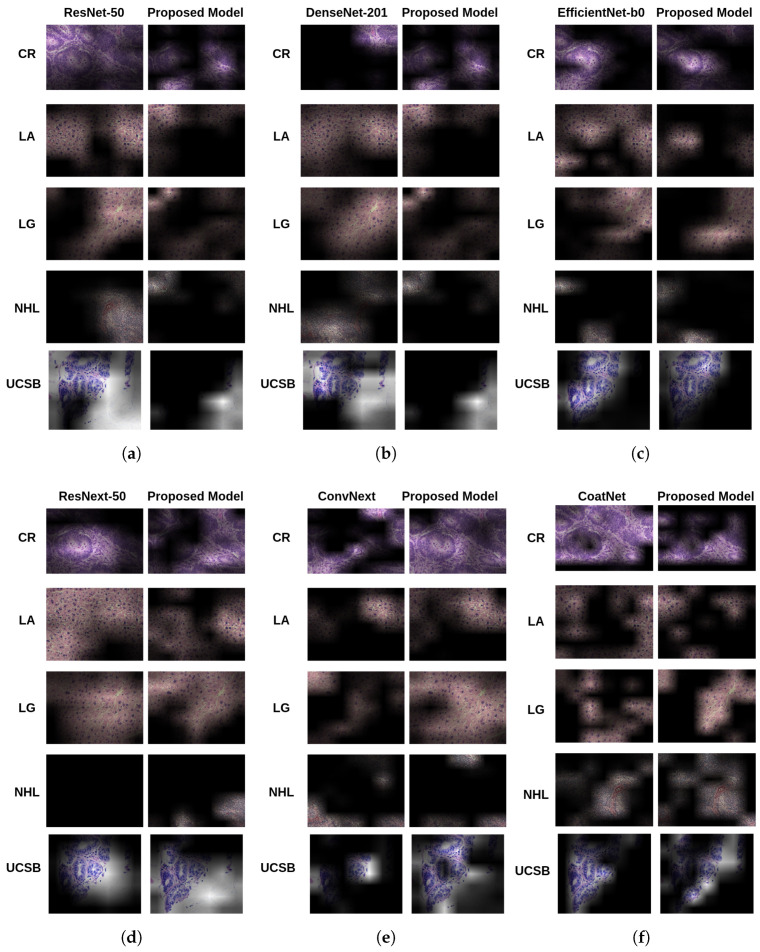
Visual comparison of Grad-CAM heatmaps produced by baseline (**left**) and proposed (**right**) models. Rows correspond to different architectures: (**a**) ResNet-50, (**b**) DenseNet-201, (**c**) EfficientNet-b0, (**d**) ResNeXt-50, (**e**) ConvNeXt, and (**f**) CoatNet-small.

Figure 4 provides a qualitative comparison of Grad-CAM explanations generated by the original and proposed models for each backbone. For each architecture, one representative image is selected, enabling visual inspection of interpretability enhancements achieved through attention supervision and CAM Fostering.

Across most backbones, the proposed models produce explanations that are more spatially concentrated, semantically aligned, and diagnostically relevant. This improvement is particularly evident in regard to ResNet-50 and ResNeXt-50, where the baseline models display diffuse and inconsistent attention across large image areas. With the proposed architecture, attention maps become focused on class-relevant tissue regions, enhancing interpretability without compromising spatial resolution. In addition, quantitative gains reinforce these visual observations. ResNeXt-50, for instance, saw an ADCC increase of 17.96% on the NHL dataset. This is consistent with visual improvements, where attention maps clearly delineate tumor morphology and class-discriminative patterns which were previously fragmented or ambiguous.

In this context, CoatNet-small exhibited the most prominent visual improvement. In its baseline form, its explanations often covered broad and imprecise areas. With the proposed enhancements, the model concentrated its activations on histologically meaningful nuclei patterns, which is particularly important for lymphoma diagnosis. This refinement aligns with its superior ADCC of 77.90% on NHL and highest overall average ADCC (67.71%), reinforcing the role of architectural synergy between convolutional and transformer components. Furthermore, the enhanced CoatNet explanations displayed greater coherence and compactness. For example, in the NHL dataset, the model achieved 40.05% coherence and low complexity values. These properties indicate explanations that are not only visually interpretable, but also robust to visual artifacts and variability, a critical consideration in clinical workflows.

In contrast, EfficientNet-b0 presented a more nuanced picture. Although its baseline performance was strong on simpler datasets (for instance, UCSB), it underperformed in more heterogeneous settings. The proposed enhancements did not lead to substantial visual gains, and in some cases slightly degraded interpretability. For example, the model exhibited a high confidence drop (67.82%) on the LA dataset, suggesting difficulty in reasoning over distributed patterns, likely due to its compact design and lack of long-range context modeling. Nevertheless, EfficientNet’s explanations remained clean and less noisy, with COM values consistently under 0.16 and relatively high coherence on datasets like UCSB. These results underscore a trade-off between model efficiency and interpretability flexibility: while EfficientNet offers stability, it may lack the architectural depth to benefit fully from entropy-driven refinement.

From these visual and quantitative analyses, it is demonstrated that the proposed model systematically improves interpretability, particularly in deeper or hybrid architectures. By aligning spatial attention with entropy-aware supervision, the generated heatmaps become more localized, discriminative, and clinically meaningful. This establishes the model as a valuable tool for histological image interpretation, supporting both predictive accuracy and transparency, key requirements for deployment in real-world medical diagnostics.

### 3.4. Classification Performance: An Overview

Although the primary objective of this work is to enhance the interpretability of deep learning models through architectural modifications, it is also important to assess whether these changes impact classification performance. Table 13 presents an overview of the average F1-score and accuracy (%) across all datasets for each backbone, highlighting the classification results achieved by the baseline models in comparison to those obtained with the proposed architecture.

The results indicate that in four out of six backbones, the proposed model either maintained or improved classification performance. The most notable gains were achieved by ConvNeXt, which recorded an increase of 3.59% in F1-score and 4.58% in accuracy. This improvement suggests that modern convolutional architectures, with advanced design features and greater flexibility in feature extraction, can incorporate interpretability constraints such as attention supervision and entropy-based regularization without degrading performance. Similar positive trends were observed in DenseNet-201 and CoatNet-small, with respective F1-score improvements of 1.25% and 1.32%. These results highlight that deeper or hybrid networks, especially those with richer connectivity patterns or transformer-based elements, are more resilient to the regularization constraints imposed by explainability mechanisms.

EfficientNet-b0 also showed stable behavior under the proposed configuration, with marginal improvements in both metrics (+0.02% in F1-score and +0.04% in accuracy). These results indicate that compact and efficiently scaled architectures, such as EfficientNet, can accommodate explainability mechanisms without degrading classification performance. Such characteristics make models like EfficientNet particularly attractive for deployment in clinical environments with constrained hardware, where both predictive reliability and transparency are essential.

In contrast, ResNet-50 and ResNeXt-50 exhibited measurable drops in classification performance. ResNet-50 showed the most pronounced decline (−7.68% in F1-score and −9.83% in accuracy), followed by ResNeXt-50 (−4.43% in F1-score and −5.40% in accuracy). These results suggest that classical convolutional architectures such as ResNet-50 and ResNeXt-50, which rely on fixed local receptive fields and lack global context modeling, may be more sensitive to the introduction of additional regularization components. Consequently, the integration of entropy-based loss terms and attention mechanisms interfered with their feature learning dynamics, highlighting the need for further adaptation or architectural refinement.

In summary, the classification performance analysis reinforces the viability of the proposed framework. In the majority of cases (four out of six backbones), predictive accuracy was either preserved or improved. Even in architectures where degradation occurred, the trade-off can be acceptable given the substantial gains in interpretability. These findings confirm that the proposed architecture not only enhances transparency in decision-making, but also maintains competitive performance in classification tasks, supporting its applicability in clinical scenarios where both diagnostic accuracy and model explainability are critical, particularly in histopathology.

## 4. Conclusions

This study introduced a modular neural architecture that integrates an attention branch mechanism with the CAM Fostering entropy-based regularizer to enhance explainability in histopathological image classification. Through comprehensive experiments on six backbone models (ResNet-50, DenseNet-201, EfficientNet-b0, ResNeXt-50, ConvNeXt, and CoatNet-small) and five H&E-stained datasets, our method achieved consistent gains in the combined explainability metric (ADCC) for five out of six architectures, with a 15.65% relative increase for ResNet-50 and a peak ADCC of 77.90% for CoatNet-small on the non-Hodgkin lymphoma dataset, while classification performance was preserved or improved in four models.

The proposed framework delivers three main contributions: a modular design compatible with both convolutional and hybrid backbones; an entropy-aware training loss that steers attention maps away from overly narrow or diffuse patterns, yielding clearer and more reliable Grad-CAM visualizations; and a quantitative evaluation suite based on coherence, complexity, confidence drop, and ADCC metrics, enabling objective assessment of saliency maps across models and datasets.

By integrating entropy-based regularization with spatial attention supervision, our approach consistently highlights diagnostically relevant regions without compromising predictive accuracy. This work, therefore, offers a principled and practical solution to enhance transparency and trust in AI-assisted histopathological diagnosis.

### Future Work

Future research will focus on enhancing the proposed architecture by integrating ViT modules directly into the attention branch, aiming to leverage their ability to capture long-range dependencies in complex tissue structures. In addition, we plan to extend the framework to fully transformer-based backbones, such as DeiT, Swin Transformer, and ViT-Base, in order to evaluate the effectiveness of entropy-aware regularization in native self-attention models. We will also evaluate the impact of the proposed modifications on state-of-the-art models for classifying histopathological images, such as the DeepCMorph model [80]. To strengthen generalization and interpretability assessments, we also intend to expand the number of datasets, particularly by exploring the Cancer Genome Atlas Program (TCGA), and incorporate alternative explanation techniques, such as attention rollout, Score-CAM, and transformer-specific saliency methods. Finally, statistical resampling can be applied to estimate confidence intervals for key metrics and strengthen result reliability.

## Figures and Tables

**Table 1:** A summary of the five histological image datasets used in this study.

Dataset	Tissue Type	Classes	Samples	Resolution
UCSB [54]	Breast cancer	2	58	896 × 768
CR [55]	Colorectal tumors	2	165	Between 567 × 430 and 775 × 522
NHL [56]	Non-Hodgkin’s lymphomas	3	173	Between 86 × 65 and 1388 × 1040
LG [57]	Liver tissue	2	265	417 × 312
LA [57]	Liver tissue	4	529	417 × 312

**Table 2:** Explainability metrics for the ResNet-50, DenseNet-201, EfficientNet-b0, ResNeXt-50, ConvNext, and CoatNet-small models on the UCSB dataset, including coherence (CO), complexity (COM), confidence drop (CD), and ADCC.

Dataset: UCSB				
**Model**	**CO ↑**	**COM ↓**	**CD ↓**	**ADCC ↑**
ResNet-50	25.94	0.11	13.92	51.26
DenseNet-201	**35.08**	**0.11**	15.92	**63.70**
EfficientNet-b0	27.40	0.11	38.91	54.33
ResNext-50	29.11	0.11	14.36	55.57
ConvNext	25.93	0.11	**11.14**	50.99
CoatNet-small	28.65	0.11	24.64	56.49

The values highlighted in bold represent the best result for each metric among all the backbone models.

**Table 3:** Explainability metrics for the ResNet-50, DenseNet-201, EfficientNet-b0, ResNeXt-50, ConvNext, and CoatNet-small models on the NHL dataset, including coherence (CO), complexity (COM), confidence drop (CD), and ADCC.

Dataset: NHL				
**Model**	**CO ↑**	**COM ↓**	**CD ↓**	** ADCC ↑**
ResNet-50	20.53	0.07	71.13	43.35
DenseNet-201	21.42	0.07	**57.95**	42.86
EfficientNet-b0	29.14	0.07	64.34	60.22
ResNeXt-50	22.74	0.07	62.49	46.45
ConvNeXt	24.99	0.07	33.87	50.99
CoatNet-small	**34.14**	**0.07**	69.19	**70.74**

The values highlighted in bold represent the best result for each metric among all the backbone models.

**Table 4:** Explainability metrics for the ResNet-50, DenseNet-201, EfficientNet-b0, ResNeXt-50, ConvNext, and CoatNet-small models on the CR dataset, including coherence (CO), complexity (COM), confidence drop (CD), and ADCC.

Dataset: CR				
**Model**	**CO ↑**	**COM ↓**	**CD ↓**	**ADCC ↑**
ResNet-50	25.39	**0.13**	8.19	50.72
DenseNet-201	26.38	0.13	5.38	50.09
EfficientNet-b0	27.70	0.14	**19.83**	54.07
ResNeXt-50	**38.11**	0.14	8.27	**65.39**
ConvNeXt	28.38	0.13	5.34	53.68
CoatNet-small	34.38	0.14	5.19	60.81

The values highlighted in bold represent the best result for each metric among all the backbone models.

**Table 5:** Explainability metrics for the ResNet-50, DenseNet-201, EfficientNet-b0, ResNeXt-50, ConvNext, and CoatNet-small models on the LG dataset, including coherence (CO), complexity (COM), confidence drop (CD), and ADCC.

Dataset: LG				
**Model**	**CO ↑**	**COM ↓**	**CD ↓**	**ADCC ↑**
ResNet-50	28.85	0.24	42.51	53.25
DenseNet-201	26.19	0.24	31.08	50.90
EfficientNet-b0	32.14	0.24	41.66	62.33
ResNeXt-50	27.18	0.24	22.81	52.54
ConvNeXt	29.07	0.24	**6.27**	54.60
CoatNet-small	**32.43**	**0.24**	53.97	**65.44**

The values highlighted in bold represent the best result for each metric among all the backbone models.

**Table 6:** Explainability metrics for the ResNet-50, DenseNet-201, EfficientNet-b0, ResNeXt-50, ConvNext, and CoatNet-small models on the LA dataset, including coherence (CO), complexity (COM), confidence drop (CD), and ADCC.

Dataset: LA				
**Model**	**CO ↑**	**COM ↓**	**CD ↓**	**ADCC ↑**
ResNet-50	19.92	0.24	71.24	40.03
DenseNet-201	26.50	0.24	60.51	52.59
EfficientNet-b0	32.36	0.24	67.82	66.84
ResNeXt-50	24.60	0.24	52.43	52.49
ConvNeXt	25.31	0.24	**51.39**	53.75
CoatNet-small	**34.98**	**0.23**	72.33	**71.60**

The values highlighted in bold represent the best result for each metric among all the backbone models.

**Table 7:** Explainability metrics (CO, COM, CD, ADCC) for the proposed model for the UCSB dataset.

Dataset: UCSB				
**Model**	**CO ↑**	**COM ↓**	**CD ↓**	**ADCC ↑**
ResNet-50	28.56	0.10	55.56	56.36
DenseNet-201	14.67	**0.09**	28.96	28.18
EfficientNet-b0	32.28	0.11	31.50	61.86
ResNeXt-50	29.03	0.11	55.56	58.12
ConvNeXt	**35.42**	0.11	**7.00**	**62.69**
CoatNet-small	27.89	0.11	22.04	55.80

The values highlighted in bold represent the best result for each metric among all the backbone models.

**Table 8:** Explainability metrics (CO, COM, CD, ADCC) for the proposed model for the NHL dataset.

Dataset: NHL				
**Model**	**CO ↑**	**COM ↓**	**CD ↓**	**ADCC ↑**
ResNet-50	35.96	0.07	71.43	73.06
DenseNet-201	27.30	0.07	65.86	57.97
EfficientNet-b0	24.63	0.07	64.48	50.94
ResNeXt-50	31.00	0.07	64.36	64.46
ConvNeXt	34.62	0.07	**0.52**	60.74
CoatNet-small	**40.05**	**0.07**	60.36	**77.90**

The values highlighted in bold represent the best result for each metric among all the backbone models.

**Table 9:** Explainability metrics (CO, COM, CD, ADCC) for the proposed model for the CR dataset.

Dataset: CR				
**Model**	**CO ↑**	**COM ↓**	**CD ↓**	**ADCC ↑**
ResNet-50	31.74	0.13	**5.74**	58.11
DenseNet-201	31.05	0.13	17.44	58.33
EfficientNet-b0	23.05	0.14	20.54	47.40
ResNeXt-50	**34.12**	**0.13**	14.13	62.77
ConvNeXt	32.41	0.13	9.98	58.03
CoatNet-small	32.20	0.13	20.73	**60.59**

The values highlighted in bold represent the best result for each metric among all the backbone models.

**Table 10:** Explainability metrics (CO, COM, CD, ADCC) for the proposed model for the LG dataset.

Dataset: LG				
**Model**	**CO ↑**	**COM ↓**	**CD ↓**	**ADCC ↑**
ResNet-50	28.54	0.24	42.50	55.08
DenseNet-201	28.96	0.24	40.88	59.81
EfficientNet-b0	32.20	0.24	43.38	63.22
ResNeXt-50	31.59	0.24	40.00	58.00
ConvNeXt	30.89	0.24	**8.53**	57.69
CoatNet-small	**37.71**	**0.24**	32.25	**69.14**

The values highlighted in bold represent the best result for each metric among all the backbone models.

**Table 11:** Explainability metrics (CO, COM, CD, ADCC) for the proposed model for the LA dataset.

Dataset: LA				
**Model**	**CO ↑**	**COM ↓**	**CD ↓**	**ADCC ↑**
ResNet-50	36.22	0.24	79.75	74.22
DenseNet-201	26.74	0.24	62.54	53.47
EfficientNet-b0	32.07	0.24	69.30	66.30
ResNeXt-50	30.02	0.24	70.89	64.37
ConvNeXt	31.61	0.24	**15.79**	59.33
CoatNet-small	**36.57**	**0.24**	75.70	**75.11**

The values highlighted in bold represent the best result for each metric among all the backbone models.

**Table 12:** Comparison of average explainability metrics (CO, COM, CD, and ADCC) before and after applying the proposed enhancements.

Model	CO ↑		COM ↓		CD ↓		ADCC ↑	
	Baseline	Proposed	Baseline	Proposed	Baseline	Proposed	Baseline	Proposed
ResNet-50	24.13	**32.20**	0.16	**0.16**	**41.40**	51.00	47.72	**63.37**
DenseNet-201	**27.11**	25.74	0.16	**0.15**	**34.17**	43.14	**52.03**	51.55
EfficientNet-b0	**29.75**	28.85	0.16	**0.16**	46.51	**45.84**	**59.56**	57.94
ResNeXt-50	28.35	**31.15**	0.16	**0.16**	**32.07**	48.99	54.49	**61.54**
ConvNeXt	26.74	**32.99**	0.16	**0.16**	21.60	**8.36**	52.80	**59.70**
CoatNet-small	32.92	**34.88**	0.16	**0.16**	45.06	**42.22**	65.02	**67.71**

The values highlighted in bold represent the best result for each metric between the proposed model and the baseline model.

**Table 13:** Average F1-score and accuracy (%) across all datasets for baseline and proposed models.

Model	F1-Score		Accuracy	
	Baseline	Proposed	Baseline	Proposed
ResNet-50	**92.71**	85.03	**91.89**	82.06
DenseNet-201	94.95	**96.20**	93.36	**95.45**
EfficientNet-b0	95.67	**95.69**	95.01	**95.05**
ResNeXt-50	**97.70**	93.27	**97.09**	91.69
ConvNeXt	93.76	**97.35**	92.10	**96.68**
CoatNet-small	94.78	**96.10**	93.86	**94.59**

The values highlighted in bold represent the best result for each metric between the proposed model and the baseline model.

## Data Availability

Data are contained within the article.

## Abbreviations

The following abbreviations are used in this manuscript:

CNN	Convolutional Neural Network
ViT	Vision Transformer
H&E	Hematoxylin and Eosin
XAI	eXplainable Artificial Intelligence
ABN	Attention Branch Network
XCNN	eXplainable Neural Network
GAP	Global Average Pooling
CO	Coherency
COM	Complexity
CD	Confidence Drop
ADCC	Average DCC
Grad-CAM	Gradient-Weighted Class Activation Mapping
